# Myeloid expression of the anti-apoptotic protein Mcl1 is required in anti-myeloperoxidase vasculitis but myeloperoxidase inhibition is not protective

**DOI:** 10.1016/j.kint.2022.08.028

**Published:** 2023-01

**Authors:** Fernanda Florez-Barros, Siobhan Bearder, Bengt Kull, Adrian Freeman, Attila Mócsai, Michael G. Robson

**Affiliations:** 1 Department of Inflammation Biology, School of Immunology and Microbial Sciences, King’s College London, London, UK; 2 Research and Early Development, AstraZeneca, Gothenburg, Sweden; 3 Emerging Innovations, AstraZeneca, Cambridge, UK; 4 Department of Physiology, Semmelweis University, School of Medicine, Budapest, Hungary

**Keywords:** glomerulonephritis, inflammation, vasculitis

## Abstract

Antibodies to neutrophil and monocyte myeloperoxidase and proteinase 3 are a feature of anti-neutrophil cytoplasmic antibody vasculitis, a disease with significant morbidity for which new treatments are needed. Mice with a myeloid-specific deletion of the anti-apoptotic protein Mcl1 have reduced numbers of circulating neutrophils. Here, we assessed if myeloid-specific Mcl1 was required in murine anti-myeloperoxidase vasculitis and whether inhibition of myeloperoxidase was protective. In a murine model of anti-neutrophil cytoplasmic antibody vasculitis, induced by anti-myeloperoxidase antibody, mice with a myeloid-specific deletion of Mcl1 were protected from disease. They had fewer crescents, neutrophils, and macrophages in the glomeruli, lower serum creatinine levels and reduced albuminuria compared with controls. At baseline and day six after disease induction they had fewer circulating neutrophils than controls. At day six there were also fewer circulating monocytes. Myeloperoxidase inhibition with AZD5904 had no effect on histological or biochemical parameters of disease, and there was also no reduction in albuminuria at day one, two, five or seven after disease induction. These findings persisted when disease was induced without granulocyte-colony stimulating factor, which increases disease severity. A second myeloperoxidase inhibitor, AZM198, also showed no evidence of an effect, although both AZD5904 and AZM198 inhibited human neutrophil extracellular trap formation *in vitro*. Thus, our results show that while myeloid-specific Mcl1 is required in this model of anti-myeloperoxidase vasculitis, myeloperoxidase inhibition is not protective.

## Graphical abstract

**Figure:**
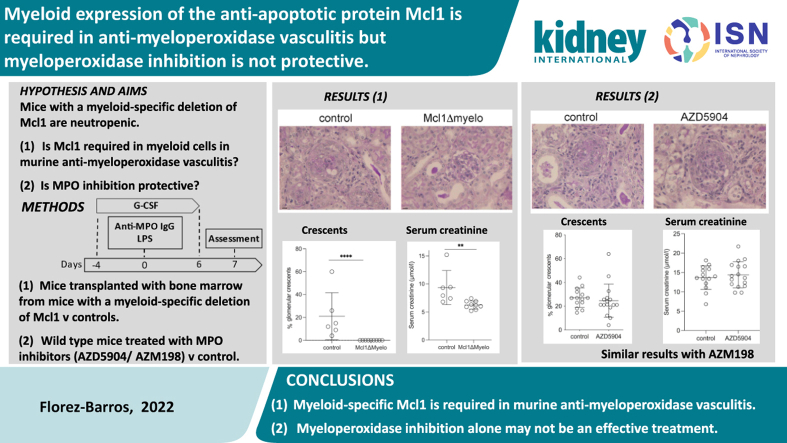

**Translational Statement**

This study suggests that myeloperoxidase inhibition alone may not be an effective treatment in anti–neutrophil cytoplasmic antibody vasculitis. Therapeutic interventions may need to target additional neutrophil or monocyte effector functions.

Anti–neutrophil cytoplasmic antibody (ANCA) vasculitis is characterized by autoantibodies against myeloperoxidase (MPO) and proteinase 3. *In vitro* studies have shown IgG from patients with anti-MPO or anti–proteinase 3 antibodies activates neutrophils.^1^ Further support for the pathogenicity of ANCA comes from *in vivo* studies in which injection of anti-MPO antibodies causes focal necrotizing crescentic glomerulonephritis in mice.^2^ We recently challenged the paradigm of neutrophil activation by ANCA^3^ by failing to demonstrate an effect on human neutrophils of MPO-ANCA or proteinase 3–ANCA from patients compared with controls using a range of assays and a large panel of IgG preparations.^4^

**Editor’s Note**

The Editors also refer you to 2 papers published on a similar topic in the November 2022 issue of *KI*: Arterial Stiffness, Endothelial Dysfunction and Impaired Fibrinolysis Are Pathogenic Mechanisms Contributing to Cardiovascular Risk in ANCA-Associated Vasculitis and Endothelium and Endothelin: Regulators of Arterial Stiffness and Fibrinolysis in ANCA-Associated Vasculitis.

Despite these findings, ANCA may have direct or indirect effects on neutrophils *in vivo* that were not evident using *in vitro* assays of activation. Therefore, we aimed to establish if neutrophils were required in ANCA vasculitis *in vivo*. A previous report in the murine anti-MPO model suggested that neutrophil depletion was protective.^5^ However, the depleting antibody used (NIMP-R14) would have also affected monocytes and so the relative importance of monocytes and neutrophils was not clear. In the current report, we used mice with a genetic deficiency of neutrophils^6^ to examine this question.

MPO is released from primary granules and is an important effector mechanism for neutrophils.^7^^,^^8^ Previous work has shown that MPO-deficient mice are protected from neutrophil-mediated glomerular injury,^9^ and delayed administration of an MPO inhibitor was effective in the autologous nephrotoxic nephritis model.^10^ Crescentic glomerulonephritis characterizes both autologous nephrotoxic nephritis and anti-MPO vasculitis. Therefore, there are likely to be common disease mechanisms and effective therapies. Mice with a myeloid-specific deletion of myeloid cell leukemia-1 (Mcl1) (Mcl1ΔMyelo) have been shown to be deficient in neutrophils.^6^ We created neutrophil-deficient mice by transplanting bone marrow from Mcl1ΔMyelo or control mice into CD45.1-positive recipients. This allowed identification of CD45.2-positive donor cells. This approach was taken because of the limited availability of Mcl1ΔMyelo mice, and to avoid the need to transfer live animals between collaborating centers. The aim of the current study was first to assess if myeloid-specific Mcl1 was required in murine anti-MPO vasculitis. If Mcl1ΔMyelo mice were protected, we then planned to assess if inhibition of MPO was protective, in keeping with findings in the autologous nephrotoxic nephritis model.^10^

## Methods

### Mice

**Figure 1:**
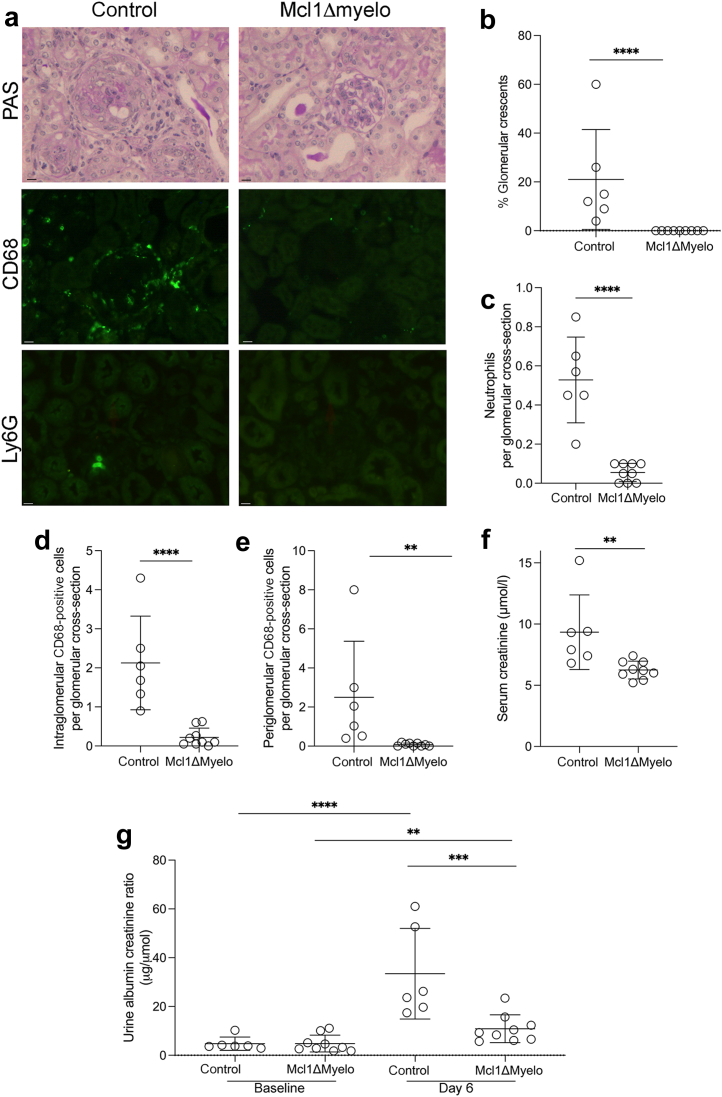
**Disease parameters in control andmyeloid cell leukemia-1 (Mcl1)ΔMyelo mice after injection of anti-myeloperoxidase antibody with glomerular colony-stimulating factor.** (**a**) Representative histology showing periodic acid–Schiff (PAS)–, CD68-, and Ly6G-stained sections from day 7. (**b**) Quantification of glomerular crescents. (**c**) Quantification of glomerular neutrophils. (**d,e**) Quantification of intraglomerular and periglomerular CD68-positive cells. (**f,g**) Biochemical parameters of disease, showing serum creatinine on day 7 and albuminuria on day 6. A 2-way analysis of variance with Šídák's multiple comparisons test was used to compare baseline and day 6 urine albumin–creatinine ratios. Day 6 urine albumin–creatinine ratios were compared using a Student's *t* test, as were the baseline values. Each symbol represents data from an individual mouse. Error bars are mean ± SD. ∗∗*P* < 0.01, ∗∗∗*P* < 0.001, and ∗∗∗∗*P* < 0.0001. Bars = 10 μm. To optimize viewing of this image, please see the online version of this article at www.kidney-international.org.

**Figure 2:**
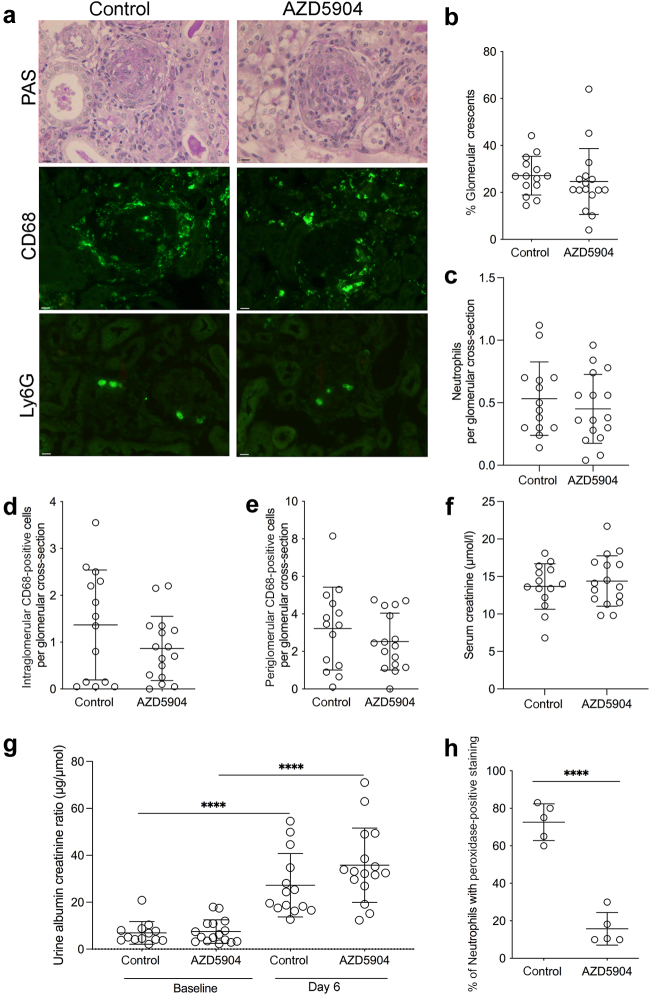
**AZD5904- or vehicle-treated mice after injection of anti-myeloperoxidase antibody with glomerular colony-stimulating factor.** (**a**) Representative histology showing periodic acid–Schiff (PAS)–, CD68-, and Ly6G-stained sections. (**b**) Quantification of glomerular crescents. (**c**) Quantification of glomerular neutrophils. (**d,e**) Quantification of intraglomerular and periglomerular CD68-positive cells. (**f,g**) Biochemical parameters of disease, showing serum creatinine on day 7 and albuminuria on day 6. Data were missing for 1 baseline urine in a control mouse for technical reasons. A 2-way analysis of variance with Šídák's multiple comparisons test was used to compare baseline and day 6 urine albumin–creatinine ratios. Day 6 urine albumin–creatinine ratios were compared using a Student's *t* test, as were baseline values. (**h**) Peroxidase staining on peripheral blood neutrophils at the end of the experiment. For (**b**)–(**g**), data are pooled from 3 experiments. For (**h**), data correspond to 1 experiment. Each symbol represents data from an individual mouse. Error bars are mean ± SD. ∗∗*P* < 0.01, ∗∗∗*P* < 0.001, and ∗∗∗∗*P* < 0.0001. Bars = 10 μm. To optimize viewing of this image, please see the online version of this article at www.kidney-international.org.

**Figure 3:**
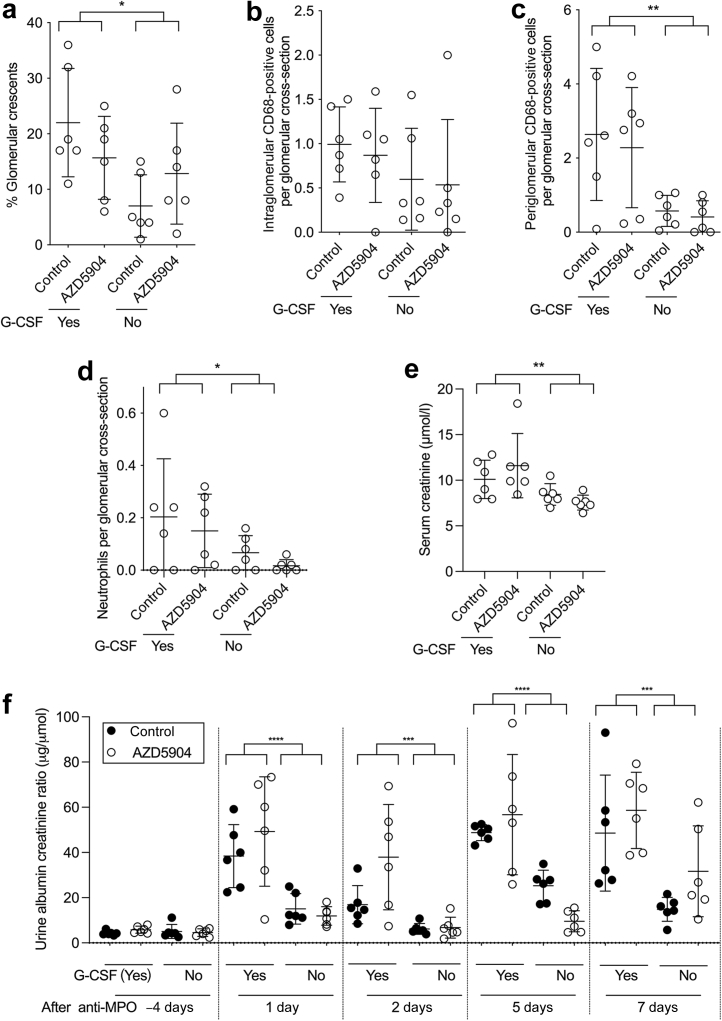
**AZD5904- or vehicle-treated mice after injection of anti-myeloperoxidase (MPO) antibody, with or without glomerular colony-stimulating factor (G-CSF).** (**a**) Quantification of glomerular crescents. (**b,c**) Quantification of intraglomerular and periglomerular CD68-positive cells. (**d**) Quantification of glomerular neutrophils. (**e**) Serum creatinine at day 7. (**f**) Albuminuria at baseline or at the indicated time point after disease induction. At day –4, groups labeled (yes) have not yet received G-CSF. Data in (**a**)–(**e**), and each time point in (**f**), were analyzed with a 2-way analysis of variance (ANOVA) and Šídák's multiple comparisons test to compare control and AZD5904-treated groups in the presence or absence of G-CSF. The ANOVA result showed that G-CSF was a significant source of variation, as indicated. Each symbol at a given time point represents data from an individual mouse. Error bars are mean ± SD. ∗*P* < 0.05, ∗∗*P* < 0.01, ∗∗∗*P* < 0.001, and ∗∗∗∗*P* < 0.0001.

**Figure 4:**
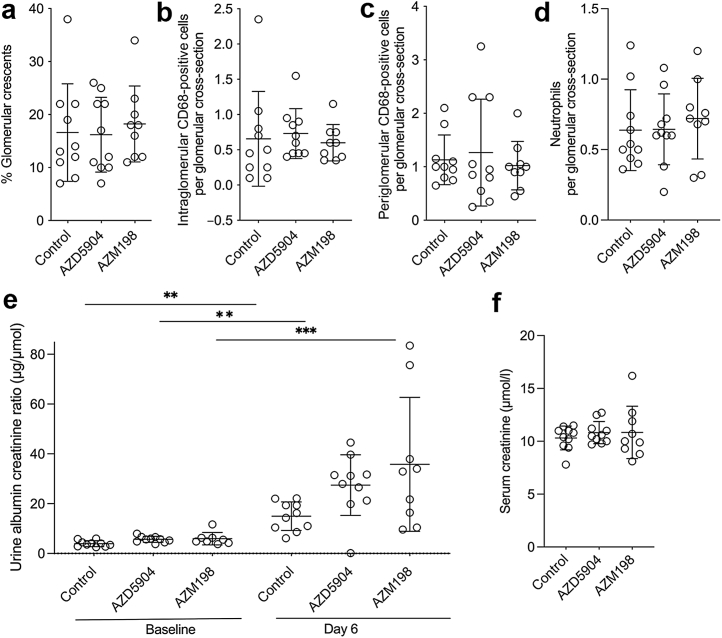
**AZD5904-, AZM198-, or vehicle-treated mice after injection of anti-myeloperoxidase antibody with glomerular colony-stimulating factor.** (**a**) Quantification of glomerular crescents. (**b,c**) Quantification of intraglomerular and periglomerular CD68-positive cells. (**d**) Quantification of glomerular neutrophils. (**e,f**) Biochemical parameters of disease, showing day 7 serum creatinine and day 6 albuminuria. Data for 1 baseline urine are missing from the AZM198-treated group for technical reasons. Data in (**a**)–(**d**) and (**f**), and the day 6 urine albumin–creatinine ratios in (**e**) were analyzed with a 1-way analysis of variance (ANOVA) and Dunnett's multiple comparisons test, as were the baseline urine albumin–creatinine ratios. A 2-way ANOVA with Šídák's multiple comparisons test was used to compare baseline and day 6 urine albumin–creatinine ratios. Each symbol represents data from an individual mouse. Error bars are mean ± SD. ∗∗*P* < 0.01, ∗∗∗*P* < 0.001, and ∗∗∗∗*P* < 0.0001.

C57BL/6 mice congenic for CD45.1 (B6. SJL-*Ptprc*^*a*^ *Pepc*^*b*^/BoyJ) were obtained from the Jackson Laboratory and bred in house. Male mice, aged 8 weeks, were used as bone marrow recipients. LysM^cre/cre^Mcl1^flox/+^ were bred in Semmelweis University, to generate Lyz2^Cre/Cre^Mcl1^flox/flox^ or Lyz2^Cre/Cre^Mcl1^flox/+^ control mice.^6^ Male mice were used as bone marrow donors at 9 weeks old, and chimeric experimental mice were called Mcl1ΔMyelo and controls, respectively (Figure 1). Female C57BL6/J mice, aged 10 weeks, obtained from Scanbur BK (Sweden) were used for pharmacokinetic studies (Supplementary Material). These were performed in Sweden by AstraZeneca. Wild-type C57BL6/J mice (aged 8 weeks) were obtained from Charles River UK for other experiments. Male mice were used for the experiments shown in Figures 2 and 3, and female mice were used for the experiment in Figure 4. Age- and weight-matched mice were used in all experiments, with random allocation to experimental groups. All experiments were performed according to local and UK home office or Swedish regulations. Mice were kept in specific pathogen-free conditions with free access to food and water.

### Bone marrow transplantation

Hind legs from donor mice were sent from Semmelweis University to King’s College London, and bone marrow was extracted within 24 hours. Recipient mice were irradiated with a dose of 9 Gy using a cesium-137 source and reconstituted with 1 × 10^7^ donor cells injected i.v. on the same day.

### Anti-MPO vasculitis model

Anti-MPO antibody was raised in MPO-deficient mice, as described,^11^ and IgG was purified by protein G chromatography (Hitrap; GE Healthcare). Day 0 denotes the day when the anti-MPO IgG was injected i.v. In the experiments with Mcl1ΔMyelo mice (Figure 1), 1 of 3 experiments with AZD5904 (Figure 2), the experiment with and without granulocyte colony-stimulating factor (G-CSF) (Figure 3), and the experiment with AZM198 (Figure 4), mice were treated with 6 μg G-CSF (Neupogen; Amgen) s.c. from day –4 to day 6. Anti-MPO IgG (2 mg/20 g) was given by i.v. injection to mice on day 0. Lipopolysaccharide (*Escherichia coli*; serotype R515; Enzo Life Sciences; ALX-581-007-L002) was given at 50 μg/20 g i.p. on day 0 and day 3. In 2 of 3 experiments with AZD5904 (Figure 2), G-CSF administration was the same, but anti-MPO IgG was 1 mg/20 g given by i.v. injection with 2.5 μg/20 g lipopolysaccharide (*E coli*; serotype R515; Enzo Life Sciences; ALX-581-007-L002) given at day 0 only and in the same i.v. injection. The MPO inhibitors AZD5904 or AZM198 (AstraZeneca) were suspended in the vehicle 0.5% hydroxypropyl methylcellulose and 0.1% Tween-80 (Sigma). A dose of 180 μmol/kg of the MPO inhibitor AZD5904 (AstraZeneca) was given to wild-type C57BL6/J mice twice a day by oral gavage (for a total dose of 90 mg/kg per day) for 7 days, starting on day –1. For AZM198, 400 μmol/kg was given twice a day by oral gavage (for a total dose of 128 mg/kg per day). Controls received vehicle alone by gavage twice a day. Spot urine samples were taken at baseline and on the day indicated. Mice were killed on day 7, with blood collected from the axillary vessels under terminal anesthesia.

### Assessment of disease

Urine albumin was measured by enzyme-linked immunosorbent assay (Bethyl Laboratories). For Figures 1 and 2, urine creatinine was measured with a creatinase assay (Diazyme) based on the manufacturer's instructions and with a standard curve generated for all assays. Urine creatinine for Figures 3 and 4 and all serum creatinine values were measured using liquid chromatography with tandem mass spectrometry in the pediatric biochemistry department at the Evelina London Children's Hospital. To assess peroxidase activity in peripheral blood neutrophils, blood was collected from the axillary vessels under terminal anesthesia 12 hours after the last dose of AZD5904. Blood films were made immediately without using anticoagulant. Films were fixed and stained with Leucognost fixing mixture and Leucognost Pox (Merck Millipore). Peroxidase activity was assessed by counting the number of peroxidase-positive neutrophils. Kidneys were fixed in Bouin solution, and paraffin-embedded sections were stained with periodic acid–Schiff. A total of 100 glomeruli per animal were assessed to score the percentage of glomerular crescents. Phosphate lysine periodate fixed tissue was used for immunofluorescence with a minimum of 20 glomeruli per sample assessed in each case. Unlabeled primary antibodies used were Ly6G (clone 1A8; BioLegend) and CD68 (clone FA11; Serotec). Slides were also stained with 4′,6-diamidino-2-phenylindole (2 μg/ml). Detection was with Dylight 488–conjugated mouse anti-rat IgG (Jackson’s Immunoresearch). All slides were coded before scoring so the researcher performing the histologic assessment did not know the sample identity. MPO was measured in serum by enzyme-linked immunosorbent assay (DuoSet; Bio-Techne), according to the manufacturer’s instructions.

### Whole blood leukocyte flow cytometry

Blood samples were taken from the saphenous vein and added to EDTA-coated tubes. Total white cell counts were determined for each mouse using a hemocytometer and EDTA anticoagulated blood diluted in Turk solution. Whole blood was blocked with 1 μg/ml mouse anti-CD16/32 Fc Block (BD Biosciences) and stained with the following conjugated antibodies: CD11b (clone M1/70; BioLegend), Ly6C (clone AL-21; BD Biosciences), Ly6G (clone 1A8; BioLegend), CD45.1 (clone A20; BioLegend), and CD45.2 (clone I04; BioLegend). Samples were run on a BD Fortessa flow cytometer using FacsDiva software (BD Biosciences), and data were analyzed using FlowJo (BD Biosciences) software. Absolute numbers of neutrophils, monocytes, and monocyte subsets were calculated from the total white cell count, and the percentage of each relative to the total CD45^+^ cells on flow cytometry.

### Pharmacokinetic and dose-finding studies

Mice were given AZD5904 twice daily, as described above for the anti-MPO vasculitis model, at the doses indicated. Blood was taken from the tail vein into EDTA, and AZD5904 concentrations were measured in plasma by liquid chromatography with tandem mass spectrometry by AstraZeneca. The concentration-dependent inhibition of mouse and human MPO was assessed using a luminol assay. The experiments were performed in MEBSS buffer (pH 7.4), containing 40 mmol/L HEPES, 5.4 mmol/L KCl, 144 mmol/L NaCl, 0.8 mmol/L Na_2_HPO_4_, 1.2 mmol/L CaCl_2_, and 0.8 mmol/L MgSO_4_. A 10 mmol/L luminol stock was prepared in distilled water and further diluted in MEBSS buffer to a final concentration of 100 μmol/L. H_2_O_2_ was prepared as 1 mmol/L stock in MEBSS buffer, yielding a final concentration of 50 μmol/L after addition into the assay. AZD5904 was serially diluted in dimethylsulfoxide in a separate plate as a 100× stock solution. Human MPO (purified in house from HL60 cells) and recombinant mouse MPO (R&D Systems) were prediluted to yield a final concentration of ≈0.16 nmol/L MPO human monomer. The experiment was run by pipetting 2 μL AZD5904 and 2 μL MPO into opposite sides of wells in a 96-well Optiplate (Perkin Elmer), followed by addition of 200 μL luminol-containing MEBSS buffer and finally 10 μL H_2_O_2_-containing MEBSS buffer. Luminescense measurement (Perkin Elmer Wallac Microbeta Trilux 1450-029; 12-detector) was started directly and followed during 15 minutes, with readout every 30 seconds. Luminescence recorded ≈15 minutes after addition of H_2_O_2_ was used to calculate the 50% inhibitory concentration values.

### Neutrophil extracellular trap formation

Human neutrophils were isolated from healthy donors (ethical approval National Research Ethics Service committee London: London Bridge 09/H084/72). Peripheral blood was collected in EDTA tubes, diluted 1:1 in Hanks balanced salt solution, and peripheral blood mononuclear cells were separated with Ficoll-Paque (GE Healthcare). Red cells were lysed in the pellet using an ammonium chloride/potassium carbonate lysis buffer (Biolegend); and, after washing in Hanks balanced salt solution, neutrophils were resuspended in RPMI medium (Sigma) with 2% AB serum (Sigma) and 1% penicillin-streptomycin (Fisher Scientific). They were plated either alone or stimulated with phorbol 12-myristate 13-acetate (500 nM) (Sigma), in the presence or absence of AZD5904 and AZM198 (10 μM), followed by 2-hour incubation at 37 °C. Neutrophils were stained with Sytox green (0.5 μM) (Invitrogen) or fixed with 1% paraformaldehyde and stained with polyclonal rabbit anti-human MPO (DAKO) and monoclonal mouse anti-human elastase (clone NP57; Insight Biotechnology), with Dylight 488–conjugated goat anti-rabbit IgG (Jacksons Immunoresearch) and Alexa Fluor 647–conjugated goat anti-mouse IgG (Jacksons Immunoresearch) as secondary antibodies. Cells were counterstained with 4′,6-diamidino-2-phenylindole (1 μg/ml). Neutrophil extracellular traps (NETs) were visualized under a fluorescence microscope, and 10 consecutive pictures were taken using the 20× objective. Images were analyzed by Fiji analysis software (open source), using a threshold to exclude background staining and quantifying the cumulative area of positive Sytox Green. A NET area index was calculated for every acquired image by calculating the ratio of Sytox Green–stained area to number of nuclei. A higher NET area index denotes a larger NET area.

### Statistics

GraphPad Prism version 9 (GraphPad Software) was used to analyze the data. For comparisons involving 2 groups, a Student's *t* test was used. For >2 groups, data were analyzed as indicated in the figure legends. Data were logarithmically transformed before analysis in some cases.

## Results

### Myeloid-specific Mcl1 is essential for anti-MPO vasculitis

Bone marrow from control or Mcl1ΔMyelo mice (CD45.2 positive) was transplanted into CD45.1-positive recipients. Peripheral blood was taken to assess chimerism and numbers of circulating leukocytes during the experiment. Flow cytometry markers used were CD45.1, CD45.2, Ly6C, CD11b, and Ly6G. The gating strategy is shown for mice receiving control marrow or Mcl1ΔMyelo marrow in Supplementary Figure S1A and B. As expected, there were few Ly6G+ neutrophils seen in recipients of Mcl1ΔMyelo marrow. At baseline, CD45.2+ donor-derived neutrophils and monocytes were assessed as a percentage of total CD45+ cells (Supplementary Figure S1C and D). More than 97% of circulating neutrophils were donor derived in all mice. In addition, >98% of circulating monocytes (Ly6C+Ly6G^–^CD11b+ cells) were donor derived in all mice.

Twenty-four hours before disease induction, there were fewer neutrophils in the peripheral blood of Mcl1ΔMyelo mice and a similar number of Ly6C^hi^ and Ly6C^lo^ monocytes (gating strategies and data are in Supplementary Figure S2A–D). Control mice showed a reduction in circulating neutrophils, Ly6C^hi^ monocytes, and Ly6C^lo^ monocytes at day 1 compared with baseline (Supplementary Figure S2B–D). At day 1, there were no differences in the number of circulating neutrophils, Ly6C^hi^ monocytes, or Ly6C^lo^ monocytes when Mcl1ΔMyelo and control mice were compared. At day 6, there were again fewer neutrophils in the peripheral blood of Mcl1ΔMyelo mice compared with control mice (Supplementary Figure S2B) and there were also fewer Ly6C^hi^ and Ly6C^lo^ monocytes (Supplementary Figure S2C and D).

Mice receiving Mcl1ΔMyelo marrow were protected from disease in the anti-MPO model. Representative histology and immunofluorescence staining for CD68 and Ly6G are shown in Figure 1a. There were fewer glomerular crescents (Figure 1b), fewer glomerular neutrophils (Figure 1c), and fewer intraglomerular and periglomerular CD68-positive cells (Figure 1d and e). Biochemical measures of disease confirmed the difference with a lower serum creatinine at day 7 (Figure 1f) and less albuminuria at day 6 (Figure 1g) in Mcl1ΔMyelo mice. Overall, these data showed less disease in Mcl1ΔMyelo mice and provided evidence that myeloid-specific Mcl1 was required for disease.

### Pharmacokinetic and *in vitro* studies to establish the dose of AZ5904

To establish the dose of AZD5904 to use for the *in vivo* experiments, we performed pharmacokinetic studies. A plasma concentration of ≈1 μmol/L was achieved 6 hours after an oral dose of 180 μmol/kg, as shown in Supplementary Figure S3A. For mouse MPO, the 50% inhibitory concentration was higher than human MPO at 190 nmol/L, as shown in Supplementary Figure S3B. Therefore, 6 hours after a dose of 180 μmol/kg, the concentration was well in excess of the 50% inhibitory concentration for the luminol assay, and close to that giving 90% inhibition (Supplementary Figure S3B). This dose of 180 μmol/kg was therefore used in the experiments with the anti-MPO model.

### MPO inhibition in anti-MPO vasculitis

Having established that myeloid-specific Mcl1 was essential, we tested the effect of MPO inhibition using the MPO inhibitor AZD5904. Inhibition was started 24 hours before disease induction. Representative histology and immunofluorescence staining for CD68 and Ly6G are shown in Figure 2a. There was no difference in crescent formation (Figure 2b), glomerular neutrophils (Figure 2c), or periglomerular or intraglomerular CD68-positive cells (Figure 2d and e), in AZD5904-treated mice compared with controls. Biochemical parameters of serum creatinine at day 7 (Figure 2f) or albuminuria at day 6 (Figure 2g) were no different between groups. We showed that MPO enzyme was inhibited *in vivo* with a decrease in peroxidase staining of peripheral blood neutrophils in treated animals (Figure 2h). There was no increase in circulating MPO levels in diseased mice compared with baseline (Supplementary Figure S4), which is in keeping with findings in patients with MPO vasculitis.^10^ However, in both mice and patients, circulating anti-MPO antibody would have affected the enzyme-linked immunosorbent assay result, leading to an underestimate of total circulating MPO. Overall, these data showed that MPO inhibition had no significant effect on disease in anti-MPO vasculitis.

### MPO inhibition in the absence of G-CSF or at earlier time points

We have previously shown that administration of G-CSF leads to more severe disease in the anti-MPO model and so used this in our initial experiments. To establish if there was an effect of MPO inhibition when G-CSF was not given, we performed further experiments. Anti-MPO vasculitis was induced as before in 2 groups of mice that were given AZD5904 or control. In the same experiment, there were 2 further groups of mice with disease induced in the same way but without G-CSF. These mice were also given AZD5904 or control. Urine was collected at baseline and at days 1, 2, 5, and 7 to assess potential effects at early time points.

As anticipated, there was more severe disease in mice given G-CSF. There were significantly more glomerular crescents and periglomerular CD68-positive macrophages at day 7 in mice given G-CSF compared with those not given G-CSF (Figure 3a and c). The difference in intraglomerular CD68-positive macrophages did not reach significance (Figure 3b), but there were more glomerular neutrophils (Figure 3d) and a higher serum creatinine (Figure 3e).

At all time points from day 1 onwards, there were significant differences in albuminuria in mice given G-CSF compared with those not given G-CSF, as indicated in Figure 3f. These findings confirmed our published work on the role of G-CSF in exacerbating disease.^11^

For mice given G-CSF, none of the parameters of disease severity was improved by AZD5904 administration (Figure 3a–f), in keeping with the data in Figure 2. Furthermore, in the presence or absence of G-CSF administration, there were no differences in any histologic parameters of biochemical parameters of disease severity when mice given AZD5904 were compared with controls (Figure 3a–f). This showed that the lack of effect for AZD5904 persisted even with the milder disease seen in the absence of G-CSF.

### Head-to-head comparison of 2 MPO inhibitors

A protective effect for another anti-MPO inhibitor, AZM198, had been shown in the nephrotoxic nephritis model.^10^ Therefore, we performed a head-to-head comparison (with G-CSF) of AZD5904 and AZM198 in the anti-MPO model using the dose of AZM198 shown to be effective in nephrotoxic nephritis. The results are shown in Figure 4. These results for AZD5904 were consistent with the data in Figure 2. Furthermore, there was no suggestion of a difference in any histologic or biochemical parameters when groups treated with AZD5904 or AZM198 were compared with controls.

### MPO inhibition with either AZD5904 or AZM198 decreases human NET formation *in vitro*

**Figure 5:**
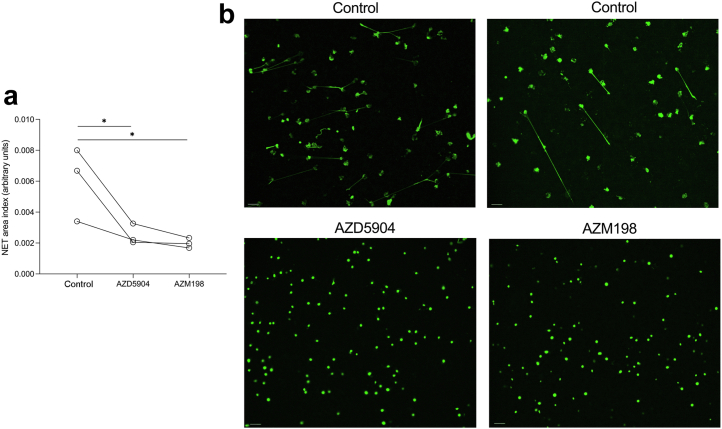
**Human neutrophil extracellular trap (NET) formation after activation with phorbol 12-myristate 13-acetate.** (**a**) Neutrophils from 3 healthy donors were treated with control, AZD5904, or AZM198. (**b**) Representative images in control and AZD5904- or AZM198-treated neutrophils. Data were analyzed with a 1-way analysis of variance and Dunnett's multiple comparisons test. ∗*P* < 0.05. Bars = 20 μm. To optimize viewing of this image, please see the online version of this article at www.kidney-international.org.

Previous work had shown that AZM198 inhibits human NET formation *in vitro*.^10^ We therefore assessed if both inhibitors had a similar effect on NET formation. We used phorbol 12-myristate 13-acetate to activate neutrophils because our previous work had shown that ANCA did not activate neutrophils compared with control IgG.^4^ Experiments were performed with neutrophils from 3 healthy donors. There was a similar reduction in NET formation seen for both AZD5904 and AZM198 (Figure 5).

## Discussion

We have previously shown, using the same neutrophil-deficient mouse strain, that myeloid-specific Mcl1 is required in the nephrotoxic nephritis model.^12^ In this previous study, there were fewer circulating neutrophils but no differences in circulating monocytes at any time point. These results suggested an essential role for neutrophils in the nephrotoxic nephritis model. In the current study, although there were no differences in monocyte subsets at either baseline or day 1, there were fewer Ly6C^hi^ and Ly6^lo^ monocytes present in the circulation of Mcl1ΔMyelo mice at day 6 compared with controls. Control mice showed a reduction in circulating neutrophils and both monocyte subsets at day 1 compared with baseline, followed by a marked increase in all cell types at day 6. The reduction in MPO-expressing neutrophils and monocytes at day 1 was probably due to depletion or recruitment to blood vessels and tissues as a result of injection of the anti-MPO antibody. However, we did not further investigate the fate of these cells at day 1. Neutrophil dysfunction can lead to secondary effects in monocytes and macrophages. For example, neutrophil-specific deletion of phospholipase Cγ2 leads to defective accumulation of monocytes/macrophages in the inflamed synovial tissue of mice with arthritis.^13^ However, this is unlikely to be the explanation for our observation of fewer monocytes in Mcl1ΔMyelo mice compared with controls at day 6. It is more likely that Mcl1 deficiency in monocytes impairs the ability of monocytes to increase in the circulation between days 1 and 6, despite the lack of a difference in baseline numbers. We did not analyze monocytes in renal tissue but would expect reduced numbers in Mcl1ΔMyelo mice, corresponding to the reduction in circulating numbers. Glomerular macrophages are derived from recruited Ly6C^hi^ monocytes, and the reduction in macrophages in and around the glomeruli may be a result of this reduction in circulating Ly6C^hi^ monocytes. In summary, protection from disease in Mcl1ΔMyelo mice is likely to be due to effects on both neutrophils and monocytes in the anti-MPO vasculitis model.

Mast cells have been shown to play a role in crescentic glomerulonephritis.^14^^,^^15^ Although Mcl1 is expressed in mast cells, we think it is unlikely that an effect on mast cells explains our results. First, this previous work in an autoimmune model of MPO vasculitis showed that mast cells enhanced autoimmunity, which is not a feature of the model we used. Second, we transplanted bone marrow from Mcl1ΔMyelo mice into irradiated wild-type recipients. As mast cells are radioresistant,^16^ wild-type mast cells would be expected to survive in the recipients and not be replaced by donor cells. Third, LysM-Cre is not active in all myeloid cells types and has been clearly shown not to activate a fluorescent Cre reporter in peritoneal mast cells.^17^ Therefore, even if some mast cells were derived from Mcl1ΔMyelo bone marrow in our experimental animals, these would be expected to express Mcl1. Overall, it is unlikely that a defect in mast cells explains our results in Mcl1ΔMyelo mice.

Our results with AZD5904 were surprising in view of the previous results for AZM198 in the nephrotoxic nephritis model. AZM198 is a related 2-thioxanthine, and both AZM198 and AZD5904 covalently attach to the haem prosthetic group of MPO. As these molecules are mechanistic siblings, we did not expect that we would obtain different results with AZM198. As anticipated, our results confirmed a lack of effect for AZM198 *in vivo*, despite a similar effect on NET formation *in vitro*. The histologic findings in the nephrotoxic nephritis model include a prominent element of glomerular thrombosis.^10^ Indeed, the effect of MPO inhibition on glomerular thrombosis was reported, but there are no data on glomerular crescents in this recent report.^10^ MPO causes endothelial dysfunction,^7^ which could promote thrombosis, and it is possible that this explains the protection seen, rather than a mechanism that would be of benefit in other forms of crescentic glomerulonephritis.

NETs have been implicated in ANCA vasculitis,^18^ and so we did not anticipate the lack of benefit from drugs that inhibit NET formation. The interventions used *in vivo* to implicate NETs included DNase treatment, receptor-interacting protein kinase 3 deficiency, and mixed-lineage kinase domain-like deficiency.^18^ These interventions may have had additional effects, and our data suggest that inhibition of NET formation may not be sufficient to inhibit disease in murine anti-MPO vasculitis. We considered if the requirement for G-CSF may obscure a potential effect of MPO inhibition. However, we confirmed that there was no benefit with AZD5904 administration when anti-MPO vasculitis was induced without coadministration of G-CSF. We also explored if there may be an early effect on disease severity that was not apparent in our previous experiments. However, there was no effect on albuminuria at day 1, 2, 5, or 7 after disease induction, in either the presence or the absence of G-CSF administration. Whatever the reasons for the differing results in the nephrotoxic nephritis model, the murine anti-MPO model of vasculitis more closely resembles ANCA vasculitis, both histologically and in terms of the disease-inducing mechanism. Therefore, it seems premature to initiate clinical studies with MPO inhibition in patients with ANCA vasculitis.

We acknowledge that we have not excluded an effect of MPO inhibition on autoimmunity. In contrast to another model of anti-MPO vasculitis,^19^ the model is not autoimmune as it relies on passive transfer of antibody. This is a limitation of the study and the model we have used. However, the pressing clinical need is for anti-inflammatory mechanisms that target the effector phase as a replacement for corticosteroids. Previous work has shown that MPO deficiency can enhance crescentic glomerulonephritis in the nephrotoxic nephritis model due to augmentation of T-cell immunity.^9^ More recent work showed that MPO inhibits dendritic cell activation.^20^ MPO inhibition may therefore enhance inflammation due to autoimmunity and is a further reason for caution in considering MPO inhibition as a treatment for autoimmune disease.

The lack of protection from MPO inhibition suggests that other neutrophil effector mechanisms may be important. Previous work has suggested that neutrophil serine proteases are required in anti-MPO vasculitis.^21^ Hence, serine protease inhibition may be a more effective therapeutic target, or there may be an effect of MPO inhibition that is revealed when it is employed alongside serine protease inhibition. Alternatively, targeting neutrophil effector mechanisms alone may not be sufficient. The protection we saw in Mcl1ΔMyelo mice may only be achievable in ANCA vasculitis if monocytes are also targeted. In conclusion, targeting myeloid effector mechanisms has therapeutic potential in patients with ANCA vasculitis, but MPO inhibition alone may not be effective.

## Disclosure

BK and AF are employees of AstraZeneca. All the other authors declared no competing interests.
