# Supplementary material for: Myeloid expression of the anti-apoptotic protein Mcl1 is required in anti-myeloperoxidase vasculitis but myeloperoxidase inhibition is not protective
Source: Kidney Int. 2023 Jan;103(1):134–43. doi: 10.1016/j.kint.2022.08.028 (PMC10166712; doi:10.1016/j.kint.2022.08.028)
Supplement: Supplementary File (PDF) [file mmc1.pdf]

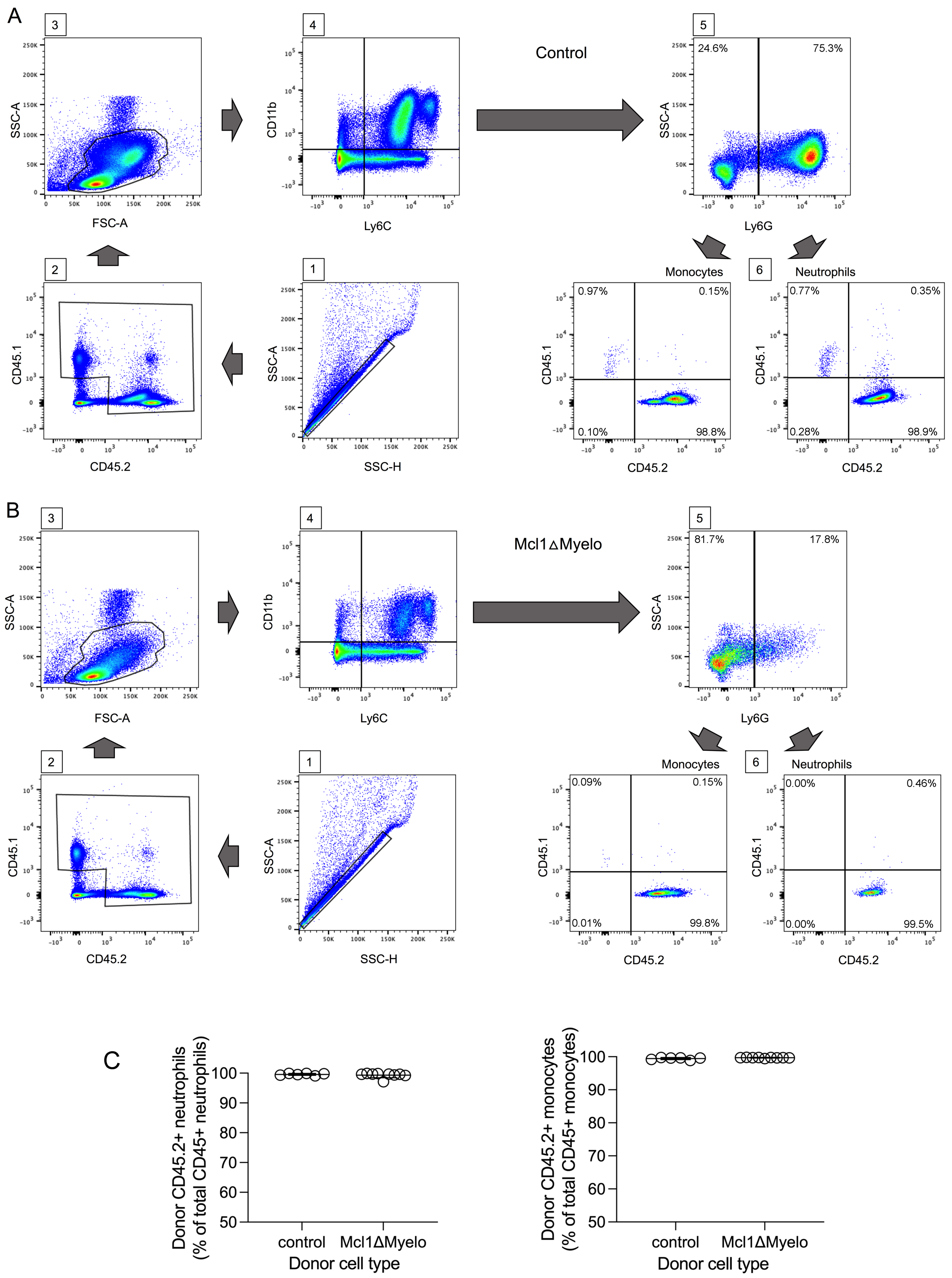

Figure S1. Circulating donor neutrophils and monocytes in CD45.1+ recipients transplanted with CD45.2+ control or Mcl1∆Myelo bone marrow. (A-B) Gating strategies for peripheral blood flow cytometry. Gate 3 defines the total CD45+ leukocyte events. Gate 5 separates neutrophils and monocytes and gate 6 defines recipient (CD45.1+) and donor (CD45.2+) cells. (C) Percentage of donor derived neutrophils or monocytes (CD45.2+) in peripheral blood at baseline. Each symbol represents data from an individual mouse. Error bars are mean ±SD.

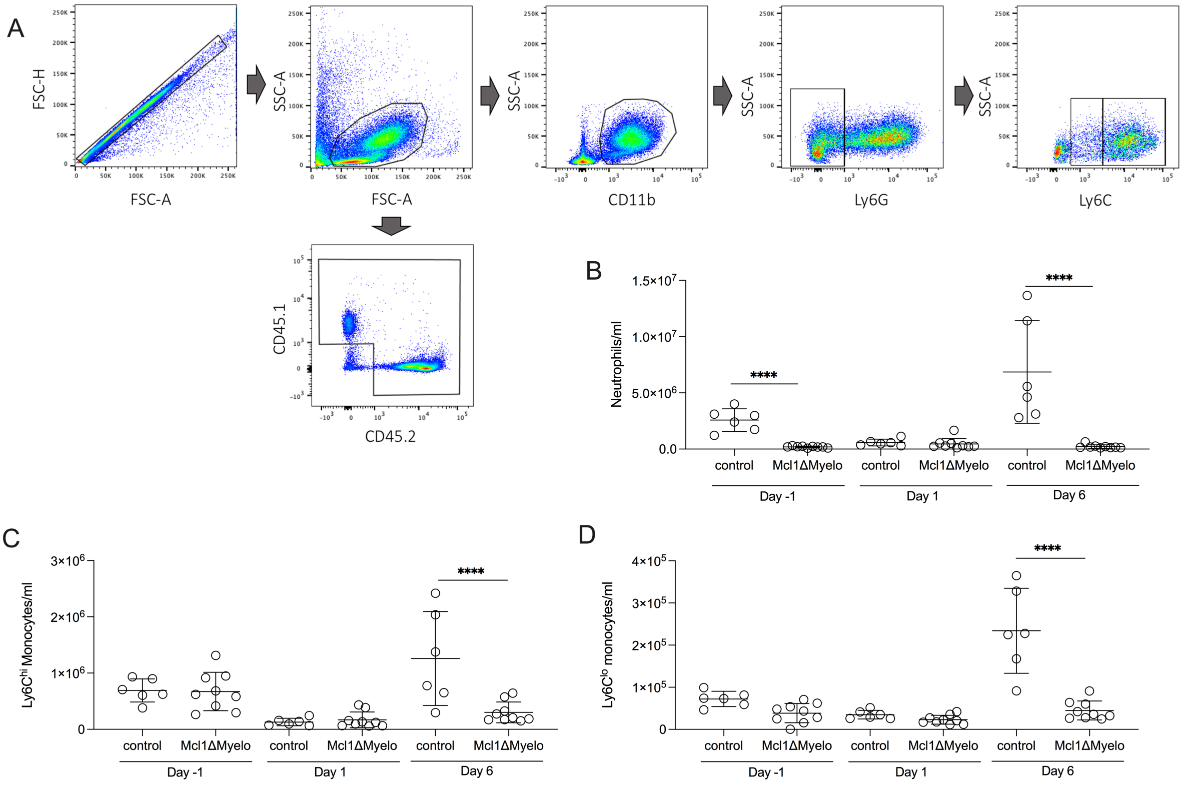

Figure S2. Circulating neutrophils and monocytes before and after disease induction. (A) Shows the gating strategy used to define neutrophils, $Ly6C^{lo}$ and $Ly6C^{hi}$ monocytes. Absolute concentrations of each were calculated from the number of counts for each cell type divided by the total CD45+ cell count (lower left panel) multiplied by the total white count (obtained with a haemocytometer). (B-D) Peripheral blood neutrophils and monocyte subsets in the two groups before and after disease induction. Data from control and Mcl1ΔMyelo mice were analysed with a two-way ANOVA and Šídák's multiple comparisons test. Each symbol represents data from an individual mouse. Error bars are mean ±SD. ****$p<0.0001$.

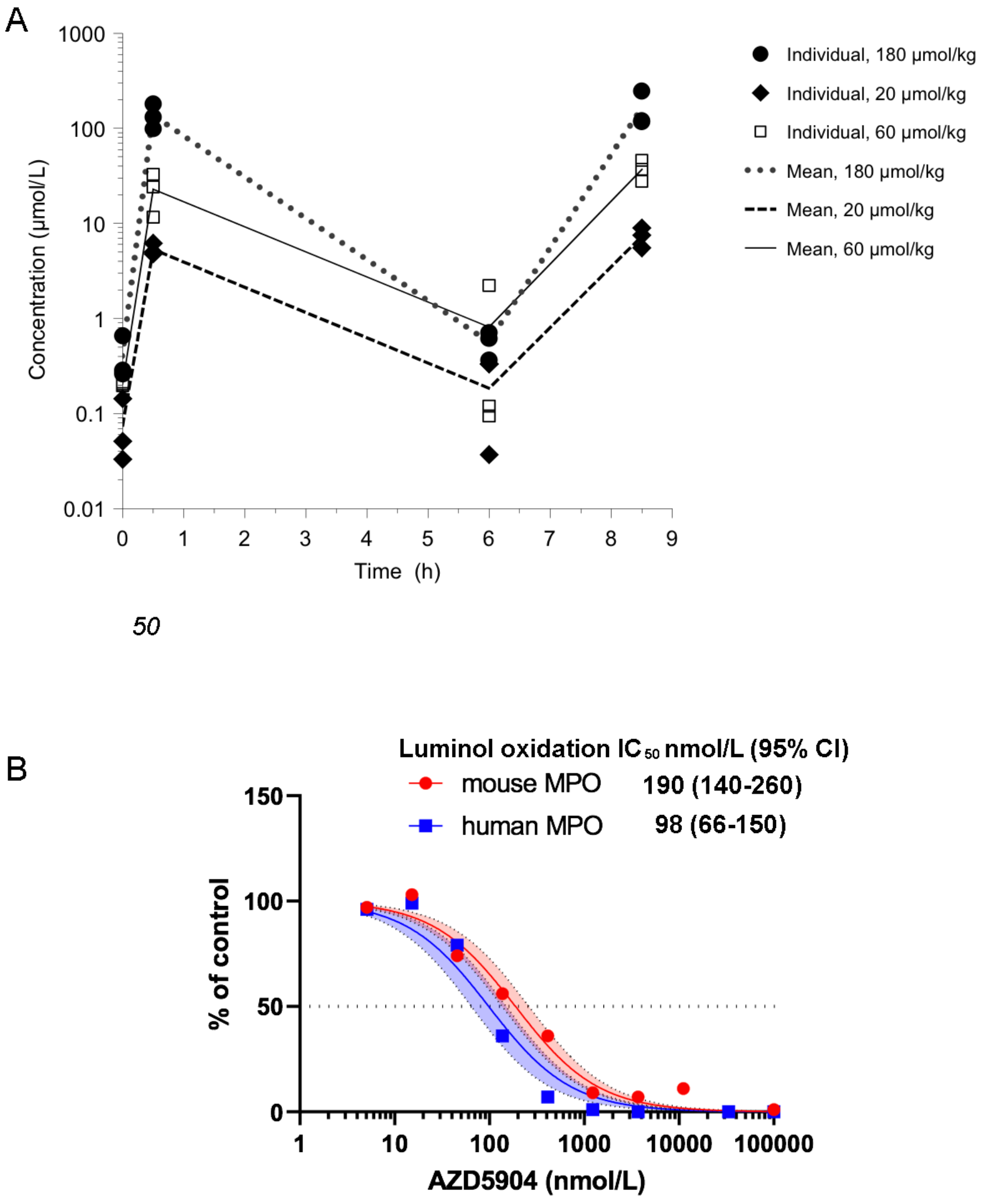

Figure S3. Pharmacokinetics and in vitro inhibitory activity of AZD5904. (A) Pharmacokinetic studies. Mice were treated twice daily with AZD5904 at the doses indicated with blood samples taken just before, 0.5 and 6 hours after the first dose. A second dose was given at 8 hours, with a fourth blood sample taken 0.5 hours later. (B) Inhibition of luminol oxidation by human and mouse MPO by AZD5904. Experiments performed under identical conditions allow a direct comparison of the IC_{50} for human and mouse MPO

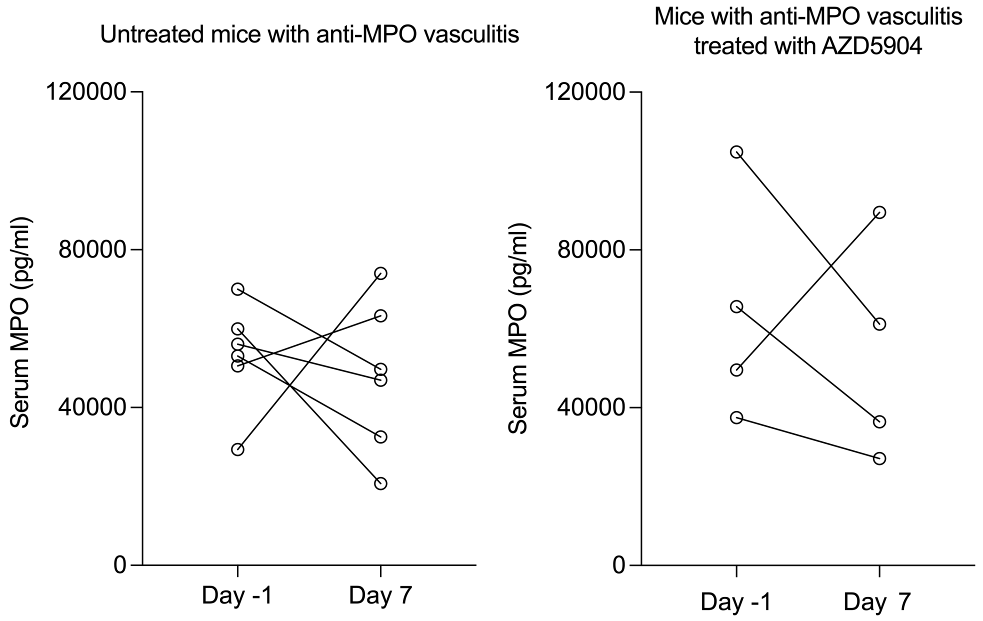

Figure S4. Myeloperoxidase levels in serum before and after disease induction. Each symbol represents data from an individual mouse.
